# An in vitro evaluation of tensile strength of molar endocrown milled from various computer aided design and manufacturing materials

**DOI:** 10.1038/s41598-024-74538-7

**Published:** 2024-10-23

**Authors:** Ahmed Ismail Taha, Aya Ehab Saad

**Affiliations:** 1 Prosthodontic Department, Faculty of Dentistry, Kafrelsheikh University, Mubark Road, Kafr Abu Tabl, 6860404, Kafrelsheikh, Kafrelsheikh Governorate 33511 Egypt; 2 Pediatric Dentistry and Public Health Department, Faculty of Dentistry, Delta University for science and technology, Mansoura, Egypt

**Keywords:** Dental materials, Prosthetic dentistry, Restorative dentistry

## Abstract

New computer-aided design and computer-aided manufacturing (CAD-CAM) materials have been reported to have suitable mechanical and physical properties for endocrown restorations. However, there is a lack of literature on evaluating the retention of endocrown using these materials. This in vitro study aimed to compare the retention of endocrowns fabricated from 3 different CAD-CAM materials. Sixty human mandibular third molars were divided into 3 groups (*n* = 20) according to the material used: (e.max CAD, Ivoclar AG, Schaan, Liechtenstein), (HC, Shofu, Koyoto, Japan), and (Brilliant Crios, Coltene, Altstätten, Switzerland) (*n* = 20). Specimens were stored in distilled water at 37 °C for 1 week and subjected to 5000 thermal cycles. A universal testing machine (5500R/1123, Instron, Norwood, USA) was used to measure the tensile force. All results were statistically analyzed using one-way ANOVA (α = 0.05), and different failure modes were demonstrated. The results revealed that e. max CAD showed the highest mean tensile force value (402.35 ± 15.812) N, and the lowest mean tensile force value was for Brilliant Coris (118.90 ± 12.430) N (*P* < .001). It is concluded that e.max CAD endocrowns showed the highest tensile force values after thermocycling in comparison to other materials with a significant difference, which may have a promising impact on the survival of endocrown restorations.

## Introduction

A post and core is the most common restoration of an endodontically treated molar. Post space is created by drilling the canal, which causes widening of root canals. These teeth usually have narrow canals and are sometimes curved with different angulations^1^. Post and core restorations usually result in 58.3% loss of tooth structure^2^, and unintended root perforations may occur^3^.

The drawbacks of post and core restoration were overcome by the introduction of endocrown restoration. Pissis was the first to fabricate an all-ceramic crown, which gained its retention from the pulp chamber and its margin rested on the cavity butt joint^2^. Thus, opposing axial walls of the pulp chamber provide macromechanical retention from^4^, while cementation with adhesive resin cement provides microretention. This conservative approach to endocrown places it as a first alternative to post and core^5^.

A lot of research supported endocrowns restorations over intra-radicular posts with direct composite resin cores as they demonstrated the same or even better clinical performance^6^. Additionally, endocrowns procedures is simple and quick, although in clinical practice, not all professionals feel confident to apply this approach^7^.

Bonding is critical for endocrown retention. The selected restorative material is one of the factors affecting the bonding of restoration, and it must be resin bonded to the tooth surface tissue^8^. In the beginning, reinforced ceramics were supported by much literature as the only material to be used for endocrown fabrication, as they demonstrated excellent adhesive properties. CAD-CAM lithium disilicate glass ceramic material as one of reinforced ceramics demonstrated both esthetic and mechanical properties with evidence in research^9^.

Many new resin-modified ceramic CAD-CAM materials that showed both resin and ceramic properties have been introduced in the market, according to manufacturers. Resin-modified ceramic material showed less brittle and more flexible behavior in comparison to conventional ceramic mentioned by Awanda and Nathanson^10^. Additionally, margins could be milled more precisely, with fewer flaws and irregularities observed, which is suitable for the minimal tooth preparation restorations. Resin nanoceramic has a special composition that shows a modulus of elasticity (12.8 GPa), like that of dentine. In addition, it does not show crack propagations as much as CAD/CAM ceramics do and has an acceptable fracture resistance rate^9^.

Adhesive failure is the most common failure mode in endocrowns^11^, because of the stress generated at the adhesive interface of glass ceramics. It was found that bonding strength to dentine is similar to that of lithium disilicate^12^. Another study concluded that lithium disilicate and composite resins are the chosen materials for endocrown, with lithium disilicate being more resistant to fracture^13^. While composite resin can be repaired in the oral cavity and does not cause wear of the opposing tooth structures as ceramic restorations^14^. In addition, a limited amount of strain can be transferred by restorative systems to the compact and spongy bone of the tooth socket^15^.

Margin integrity may also be affected by the material properties. Resin-based materials can be milled into smaller thickness because of their polymer matrix nature^16^. Unlike the ceramic glass matrix, which is brittle, crystallites of ceramic may break out easily during milling^16^. The larger the crystallites, the more likely cracks might occur^16^. Resin-based composite material restorations had been reported to have higher marginal integrity than ceramic materials^17^. However, other research demonstrated that resin composite materials had higher microleakage rates over time^9^.

The influence of the different CAD-CAM materials of endocrowns on their survival had been illustrated in literature^18,19^, but limited research was available on which material can provide better retention for endocrown restorations. Endocrown retention test designs involve cementing crowns under specific load conditions and pulling them off in an axial direction once the cement has solidified. This approach is more closely aligned to clinical testing of dental cement’s retention capabilities on different substrates^20^.

The authors are unaware of the performed studies on evaluating the influence of different materials on retention of endocrown restorations. Retention of endocrowns is a crucial factor for the survival of these restorations. Therefore, the purpose of this in vitro study was to compare retention of endocrown restorations fabricated from 3 different CAD-CAM materials: lithium disilicate glass-ceramic, resin-modified ceramic, and hybrid composite material. The null hypotheses tested that no significant difference would be found among retention values of the three groups.

## Materials and methods

This in-vitro study was approved by the ethical committee of the Faculty of oral and dental medicine and surgery, Kafrelsheikh University, Kafrelsheikh, Egypt (MKSU/22-11-3). Sixty human mandibular third molars had been extracted because of impaction problems at oral and maxillofacial surgery clinics under the approval of the local institutional review board protocol, and informed consent for using extracted teeth was obtained from patients. The inclusion criteria of selected teeth were teeth should be sound, free of caries, and without cracks. The crown diameter should be within a range of 7 to 8 mm buccolingually and 9 to 10 mm mesiodistally measured 3 mm above the cementoenamel junction (CEJ). The height of the pulp chamber was 5 to 7 mm starting from the central groove to the floor pulpal chamber and measured with a graded explorer through an access opening. The selected teeth were ultrasonically cleaned and kept, until used in the experiment, in 0.1% thymol disinfectant dissolved in distilled water at room temperature.

Selected teeth were divided into 3 groups (*n* = 20) according to CAD-CAM material used as shown in Table 1. The sample size had been determined to be adequate for a statistical power of 80% (G*Power 3.1.9.2.; Heinrich Heine University Düsseldorf)^21^: Group MX restored with lithium disilicate (e.max CAD, Ivoclar AG, Schaan, Liechtenstein), Group HC restored with resin-modified ceramic (HC, Shofu, Koyoto, Japan), and Group BN restored with hybrid composite (Brilliant Crios, Coltene, Altstätten, Switzerland) (*n* = 20).

**Table 1:** CAD-CAM materials used in the present study.

Group	Block used	Manufacturer	Composition	
MX	e.max	Ivoclar AG	Lithium disilicate glass ceramic	70 vol% lithium disilicate and glass ceramic
HC	Shofu Block HC (SH)	Shofu	Resin-modified ceramic	61% zirconium silicate, UDMA, TEGDMA, Micro fumed silica.
BN	Brilliant Crios	Coltene	Reinforced composite	29.3 wt% Cross-linked methacrylates and 70.7 wt%Amorphous silica

The pulp chamber access was drilled using a diamond bur mounted on a high-speed handpiece with copious water spray. Barbed broaches were used to remove pulpal remnants. Rotary files (protaper gold, Dentsply Sirona, Ballaigues, Switzerland) were used for root canal preparation. All selected teeth were endodontically treated in the same sequence. File F2 was the master file for the mesial canals, while file F3 was the master file for distal canals. Canals were irrigated with sodium hypochlorite. Protaper paper points and gutta percha with the same size as the master file were used. Resin-based root canal sealer (Adseal, Meta Biomed CO., Chungcheongbuk-do, Republic of Korea) was used, and the excess gutta percha was cut using a hot plugger.

A cylindrical mold was used to place each specimen centrally in a self-cure acrylic resin material (Lucitone HIPA, Dentsply Sirona, Ballaigues, Switzerland) to a level of 2 mm apical to buccal CEJ, with the help of a dental surveyor (Paraskop, Bego, Bremen, Germany) to simulate the normal biological width.

A straight handpiece was attached to the surveyor device, to which a low-speed double-sided diamond disc (NTI Serrated; Kerr Corp., Kloten, Switzerland) was attached. All specimens were decoronated 3 mm occlusal to the highest point of the pulpal floor and perpendicular to their long axis under copious water coolant. The wall of the pulp chamber was prepared with a tapered diamond rotary instrument with rounded end (TR-13-No.198/018, Mani, Osaka, Japan)^22^ held perpendicular to the floor and guided by the surveyor. At the end of preparation, all the internal angles were rounded.

A flowable resin composite (Tetric N-flow, Ivoclar AG Schaan, Liechtenstein) was applied to the floor of pulp chambers of all specimens to achieve standard pulp chamber depths of 3 mm from the sectioned molar occlusal table. The composite was cured using a polywave LED-based visible light curing (VLC) unit (Bluephase G2, Ivoclar AG Schaan, Liechtenstein).

All optical scans were performed with a wireless intraoral scanner (Medit i700, MEDIT Corp., Seoul, Republic of Korea) by a single operator (A T). Standard tessellation language (STL) file was exported and transferred to a software program (DentalCAD 3.0 Galway 2021, exocad, Darmstadt, Germany) that was used to design the endocrown restoration.

**Figure 1:**
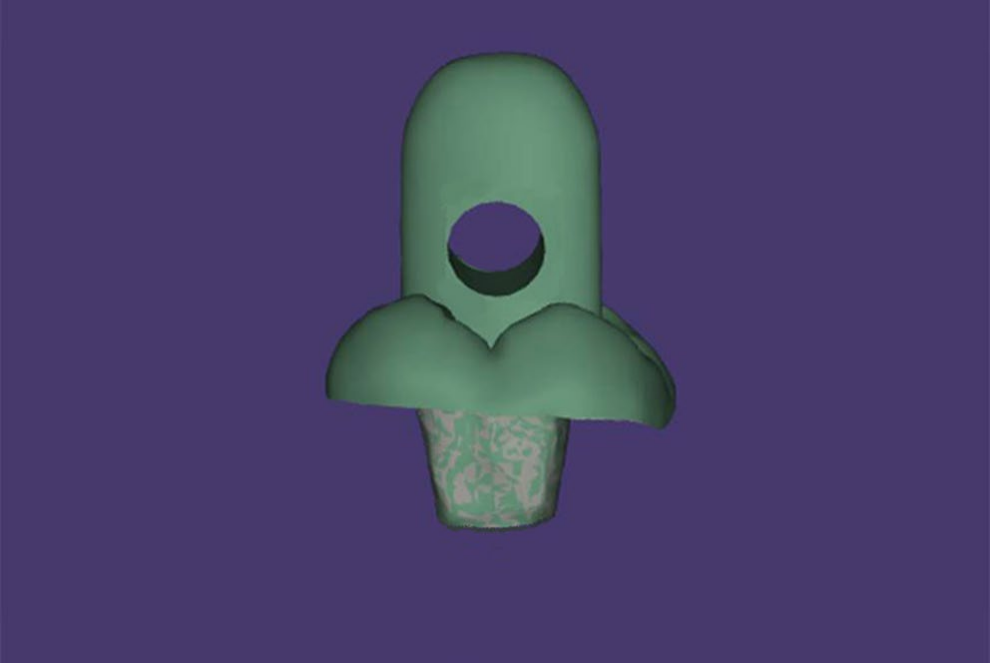
The virtual design of endocrown.

In all specimens, the coronal part of the endocrown was designed at 4 mm in height to provide consistent coronal thickness and form for the retention test. A loop ring was added to the design of the occlusal surface that helped in the retention test, as shown in Fig. 1. The occlusal surface of the endocrown was designed for a 2 mm diameter hole, which is located 2 mm above the occlusal surface and 4 mm above the hole and 2.5 mm on each side of the hole. The virtual images of the restorations were designed with a cement space of 40 μm and were transferred to a 5-axis milling machine (Coritec 250i, imes-icore GmbH, Eiterfeld, Germany).

The endocrown restorations were milled from the 3 CAD-CAM materials selected for the study. After milling, a diamond wheel (DCB, Schleifer, Komet Dental, Lemgo, Germany) was used to finish the remaining part of sprue. Endocrown restorations for group MX were placed on crystallization tray for crystallization in a furnace (Vita Vacumat 6000 M; VITA Zahnfabrik GmbH, Bad Säckingen, Germany) following the manufacturer instructions.

**Figure 2:**
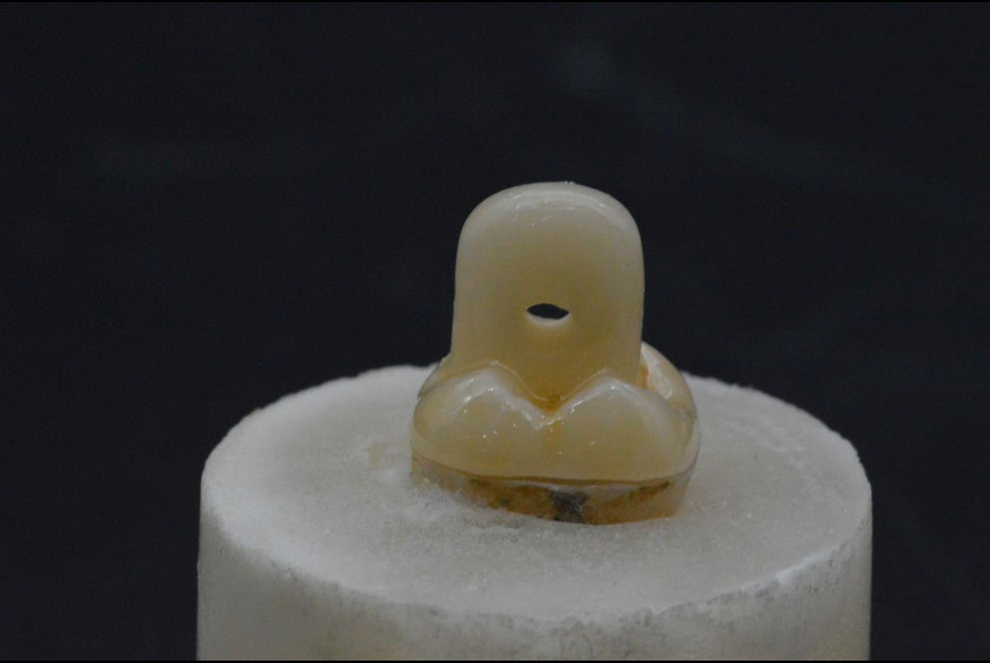
The endocrown try in after milling.

After milling, endocrown restorations were evaluated on the prepared teeth, and pressure areas were identified by using a water-soluble pressure-indicating paint (PIP; Keystone Industries, Singen, Germany). A finishing green diamond point (DCB, Schleifer, Komet Dental, Lemgo, Germany) was used to remove all detected pressure areas until complete seating was verified as shown in Fig. 2. Improvement of marginal gaps was noticed by 2 clinicians using sharp explorer at different points at the margin. Finally, cotton moistened with alcohol was used to clean all specimens and restorations.

Different protocols of surface treatment were used according to the material of the endocrown and the manufacturer’s instructions. For the MX group, hydrofluoric acid etch gel 4.5% (Porcelain etch, Ultradent Products, Cologne, Germany) was applied to the fitting surface for 20 s, rinsed, and dried, followed by application of silane coupling agent^23^. For both HC and BN groups, the fitting surface was sandblasted with 50 μm aluminum oxide (Al2O3) particles from a working distance of 10 mm for 10 s, followed by surface cleaning with phosphoric acid etchant gel 37%. Finally, primer (HC, Shofu, Koyoto, Japan) was applied for the HC group and air dried. While a universal adhesive (one coat 7, Coltene, Altstätten, Switzerland) was applied for the BN group and air dried. The enamel surface of the tooth was treated with 37.5% phosphoric acid etch (Ultra-Etch, Ultradent Products, Cologne, Germany) for 30 s, rinsed, and dried.

Endocrowns were cemented with a self-adhesive resin cement (Rely-X Unicem, 3 M ESPE, St. Paul, USA) using a constant and firm digitally applied pressure with stabilization to aid in seating of the restoration. Endocrowns margins were exposed to LED-based visible light cure (Bluephase G2, Ivoclar AG Schaan, Liechtenstein) for 2 s. Excess cement was removed, and finally, each surface received additional light curing for 20 s. After cementation, specimens were stored in distilled water at 37 °C for 1 week and then subjected to 5000 thermal cycles in water baths with both temperatures (5 °C and 55 °C); the dwell time was 30 s at each bath, and the transfer time was 10 s.

**Figure 3:**
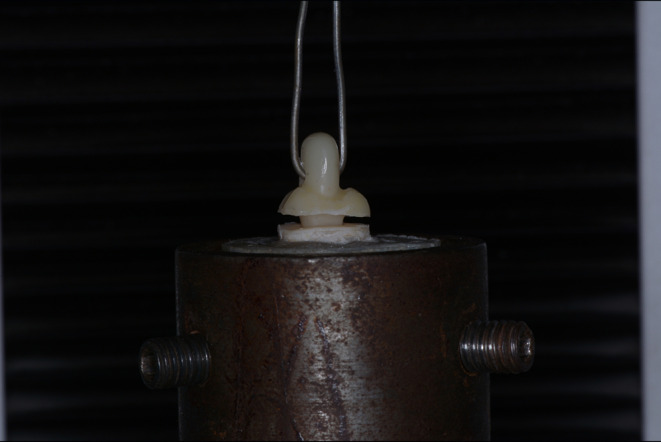
The retention test using universal testing machine.

For measuring the tensile force of cemented endocrown restoration, a universal testing machine (5500R/1123, Instron, Norwood, USA) was used. An orthodontic wire with a 1 mm cross-section was passing through the ring integrated on top of the occlusal surface and attached to the upper member of the machine as shown in Fig. 3. The other end of the sample was attached to the lower member of the testing machine. The tensile force will be applied to the samples at a cross-head speed of 0.5 mm/min and a load cell of 2 kN until failure of bond. Specimens were evaluated for the failure mode after a tensile force test. Four types of failure modes were used: type I, which referred to cohesive failure of the cement layer; type II, which referred to adhesive failure at the tooth-cement interface; type III, which referred to adhesive failure at the restoration-cement interface; and type IV, which referred to fracture of the restoration without debonding.

The values of tensile force were collected and tabulated. Data were analyzed using ANOVA test to compare between the paired groups, followed by Post-hoc Tukey test. The three groups were compared by ANOVA one-way test followed Post-hoc Tukey test. Data were statistically analyzed with a statistical software program (IBM SPSS Statistics, v20.0, IBM Corp., New York, USA). Normality of quantitative data was initially tested using the Shapiro-Wilk test, with data being normally distributed if *P* > .050. Inspecting boxplots was used for testing the presence of significant outliers (extreme values). Quantitative data were expressed as mean ± standard deviation (normally distributed). Qualitative data were expressed as N (%).

## Results

Table 2 presented data as mean ± standard deviation as well as standard error, 95% CI for mean and range (minimum and maximum).

**Table 2:** Tensile forces (newton) in the three study groups.

Group	*N*	Mean ± SD	SE	95% CI for mean		Minimum	Maximum
				Lower	Upper		
MX	20	402.35 ± 15.812	3.536	394.95	409.75	377	432
HC	20	250.45 ± 11.834	2.646	244.91	255.99	228	270
BN	20	118.90 ± 12.430	2.780	113.08	124.72	101	148

Notes: SD = standard deviation. SE = standard error. CI = confidence interval.

**Figure 4:**
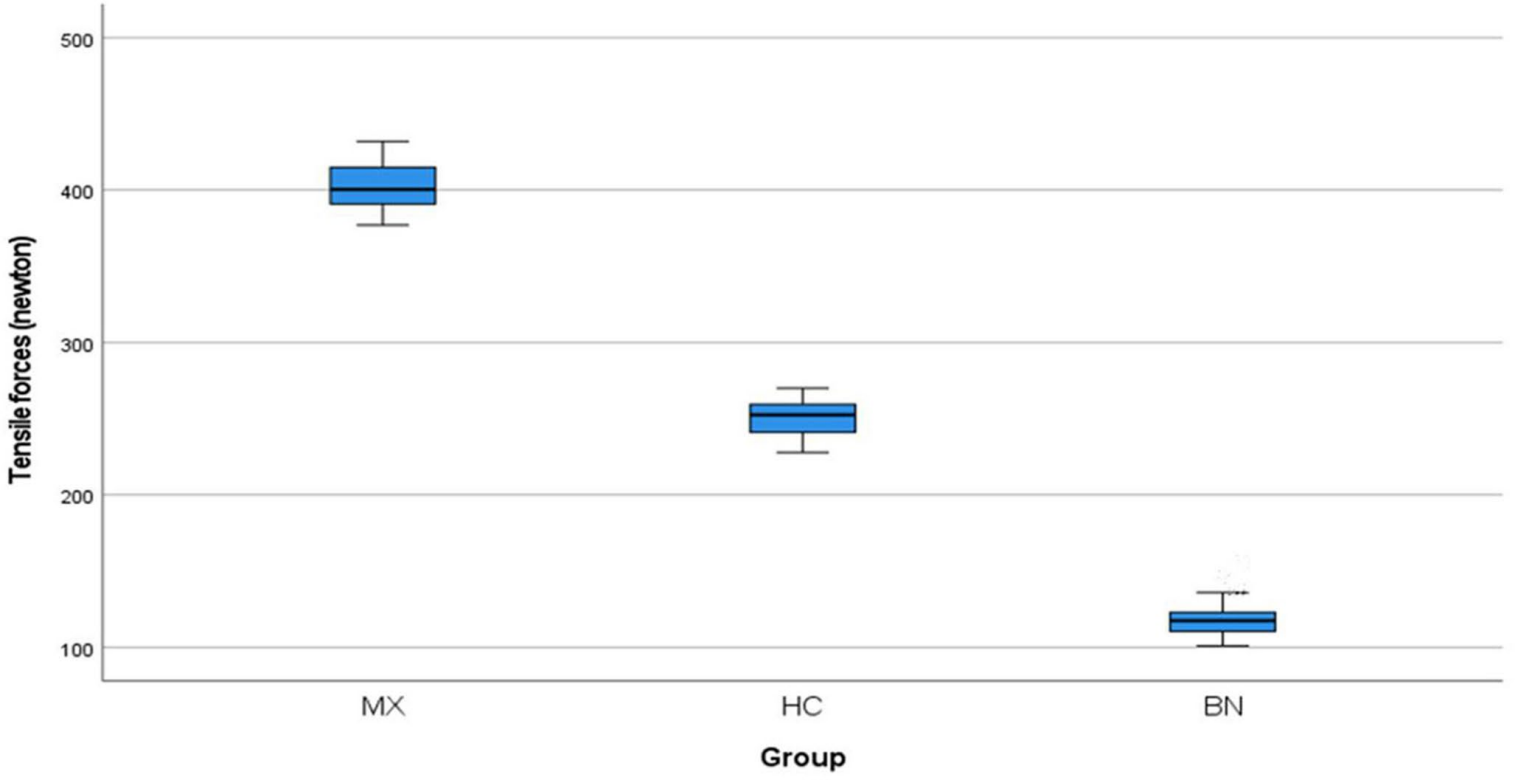
Boxplot for mean tensile forces (newton) for different CAD-CAM materials.

Table 3 showed a statistically significant difference in tensile force failure (newton) between 3 groups. Group MX showed the highest mean tensile force value (402.35 ± 15.812) N, followed by group HC (250.45 ± 11.834) N, and the lowest mean tensile force value was for group BN (118.90 ± 12.430) N (*P* < .001) as shown in Fig. 4.

**Table 3:** One-way ANOVA for tensile forces (newton).

	Sum of squares	df	Mean square	F	Sig.	η^2^
Between Groups	804819.4	2	402409.717	2216.7	0.000	0.987
Within Groups	10347.3	57	181.532			
Total	815166.7	59				

Notes: df = degrees of freedom. Sig. = p-value. η^2^ is a measure of effect size.

**Figure 5:**
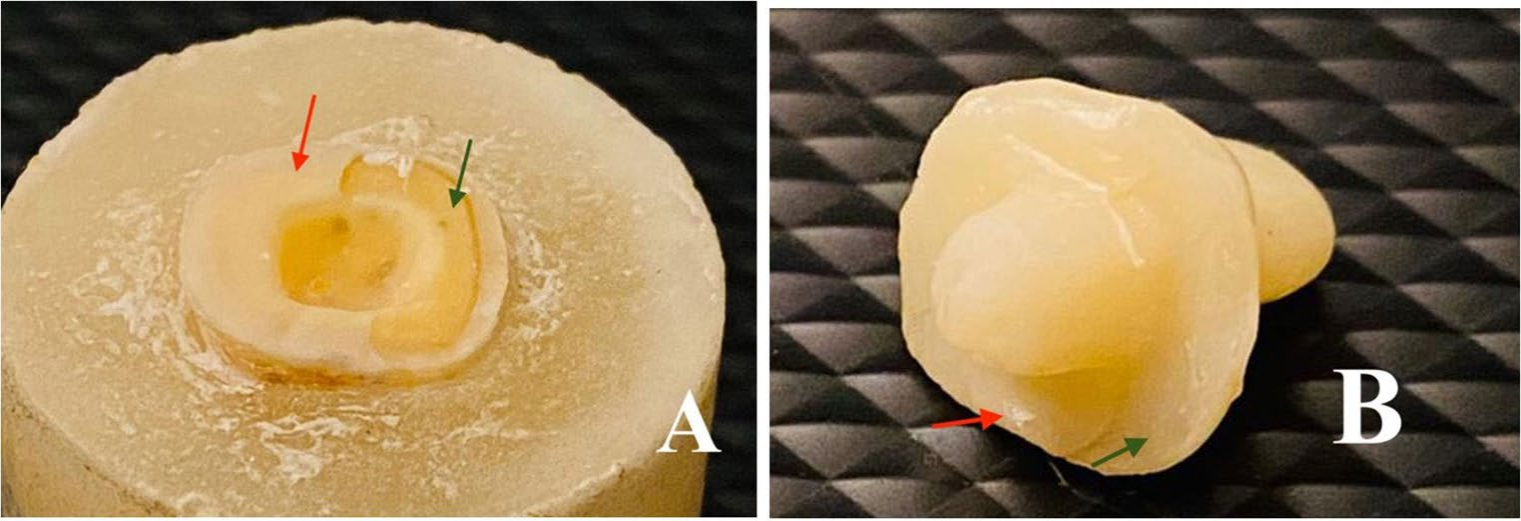
**A** and **B**, Example of failure mode type I (cohesive failure) in group MX (The red arrow marks areas where cement adheres, while the green arrow marks the area clear of cement).

**Figure 6:**
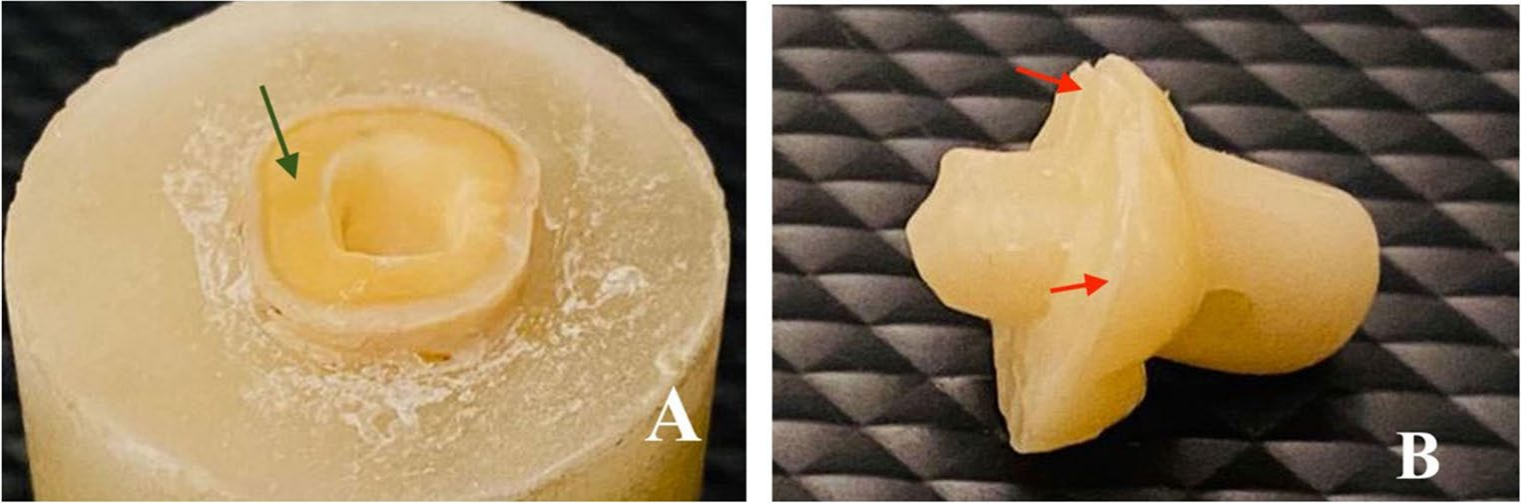
**A** and **B**, Example of failure mode type II in group MX (The red arrow marks areas where cement adheres, while the green arrow marks the area clear of cement).

**Figure 7:**
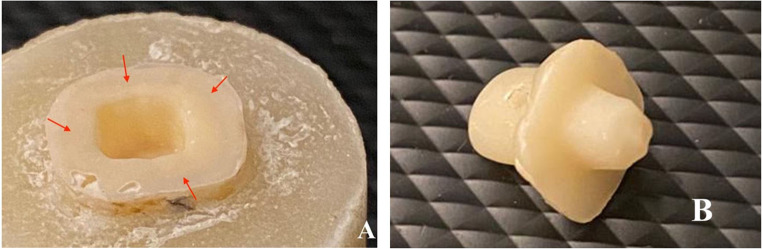
**A** and **B**, Example of failure mode type III in group HC (The red arrow marks areas where cement adheres, while the green arrow marks the area clear of cement).

**Figure 8:**
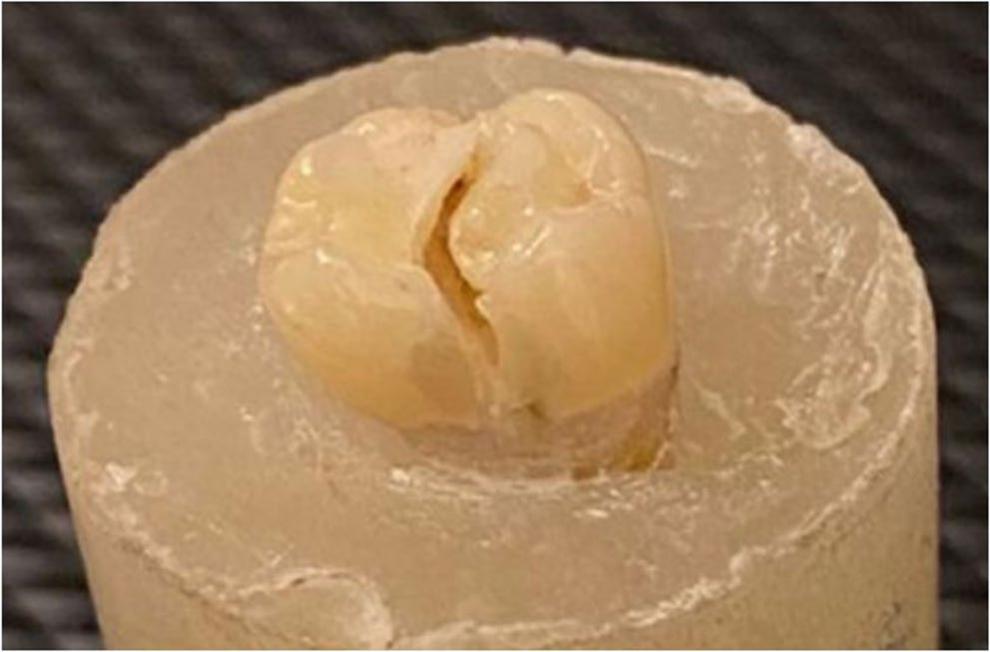
Example of failure mode type IV in group BN.

Table 4 showed that endocrowns in group MX mostly failed by type I and type II failure modes equally. In group HC, endocrowns were often failed by type III failure mode. While in group BN, endocrowns mostly failed by type IV failure mode, as shown in Figs. 5, 6, 7 and 8.

**Table 4:** Four different failure modes incidence in 3 groups.

Groups	Failure modes			
	Type I	Type II	Type III	Type IV
MX	8	8	0	4
HC	2	0	18	0
BN	4	4	0	12

## Discussion

Fracture resistance and retention are considered the most prominent factors in the survival of conservative restorations^24^. Considering the environment of the oral cavity, compressive, tensile, and shear forces intermittently and frequently affect the restoration, which may cause debonding at the interface between the tooth and the restoration through time, leading to the loss of restoration retention^24^.

This study aimed to compare the tensile force values of endocrowns constructed from three CAD-CAM materials: IPS e.max CAD, HC Shofu, and Brilliant Coris blocks. The null hypothesis was rejected, since lithium disilicate had the highest tensile force value with a statistically significant difference compared to resin-modified ceramic and reinforced composites (*P* < .001) which were easily debonded at much lower force.

The difficulty of pulling out ceramic restorations from the underlying tooth structure without the tooth or ceramic restoration being fractured is very common^25^. Previous research reported fracture of a pull-out loop integrated in the occlusal surface of fabricated crowns during testing^26^. For this reason, the thickness of the loop used in this study was increased to withstand debonding forces without fracture. Additionally, the occlusal thickness of 4 mm was intended to provide bulk material to support retention tests without fracture. According to Mörmann et al., ceramic endocrowns with a traditional preparation and an occlusal thickness of 1.5 mm had a 50% worse fracture resistance than endocrowns with a 5.5 mm occlusal thickness^27^. To simulate the clinical situation, thermocycling was done 5000 cycles with bath temperatures of 5 °C and 55 °C and dwell time of 10s to simulate the temperature changes of an oral cavity under 6 months of clinical service^28,29^.

Relyx unicem-self-adhesive universal resin cement was used in this study, as it has been increasingly used because of the simplicity of single-step cementation, low technique sensitivity, and reduced postoperative sensitivity^30–32^. In addition, to overcome some disadvantages of both classical cements (such as zinc phosphate and zinc polycarboxylate) and conventional resin cements. It combines both the advantages of classical cements for being handled easily and conventional resin cement for having the highest bonding strength, mechanical properties, and esthetics. It has a simple bonding procedure because of the acidic monomers included in their structure without additional use of etchant and/or primer/ adhesive^33^. Although, when compared to conventional resin cement systems, self adhesive resin cement showed equivalent or lower bond strength values in bonding to dentin or enamel, respectively^34,35^. However, self-adhesive resin cements were selected for bonding endocrown restorations because of the mentioned advantages and according to a similar previous study by Fages and Bennasar^36^.

Regarding adhesive cementation, the meeting surfaces need pretreatment; the dental substrate (enamel and dentin) and the fitting surface of restorations^37^. With respect to the tooth surface, the enamel part was treated with phosphoric acid etch, which dissolves the hydroxyapatite crystals, resulting in micro-irregularities into which resin infiltrates and forms resin tags, which help in micromechanical retention, thus improving adhesion properties^38^.

Concerning the fitting surface of lithium disilicate endocrown restorations, it is composed of two main components: the glassy phase (silica), which provides good bonding distinctive to adhesive resin, and about 70% lithium oxide crystals, which have the advantage of high mechanical properties^39^. Surface etching of lithium disilicate with hydrofluoric acid for 20 s provided a roughness in the surface, which increased bonding to the resin cement. A silane coupling agent was applied to form a chemical bond between the resin cement and lithium disilicate material to increase the bond strength of the restorations^40^. Accordingly, the structure of fully crystallized lithium disilicate ceramics is important to create a strong bond, which was the cause of the highest tensile force value in this study. This result was consistent with previous studies^41,42^, which showed that the highest retentive values were reported for lithium disilicate restorations cemented with self-adhesive resin luting agent.

In respect of both resin-modified ceramic (HC Shofu) and reinforced composite (Brilliant Coris), the intaglio surface was treated according to manufacturer recommendations to improve the bond strength. Both materials were surface treated by sandblasting using 50 μm aluminum oxide (AL2O3) particles, cleaned with phosphoric acid etchant gel 37%, and followed by HC primer application for HC Shofu and one coat 7 universal adhesive for Brilliant Coris.

Considering HC Shofu endocrowns, it showed lower tensile force values than that of lithium disilicate endocrowns. That result is consistent with the explanation of a previous study^43^ that demonstrated the primer agent alone may be insufficient for bonding to non-silica-based filler particles like zirconia. In addition to the lack of chemical bonds mentioned by the manufacturer, the role of HC Primer infiltrates the polymer matrix and creates micro-mechanical retention between primer and restoration.

In the case of Brilliant Coris, during early tensile load, surface fatigue cracks were formed, which can cause deterioration of composite material, which significantly decreases the capability of the composite to withstand against the tensile forces and propagates the sequences of catastrophic fracture type IV^44^. Thermocycling effect on composite endocrown was a second reason for explaining why most composite endocrowns fractured before debonding, which did not represent the actual tensile force of Brilliant Coris endocrowns.

The failure mode of resin-modified ceramic was mostly an adhesive failure because the endocrowns were debonded from the tooth structure without damaging the supporting tooth structure. Nevertheless, lithium disilicate ceramic endocrowns demonstrated adhesive and cohesive failure, as lithium disilicate is shown to have the highest bond strength to adhesive resin cement. Brilliant Coris mostly showed cohesive failure type IV with restoration fracture because of tensile forces and the thermocycling effect. According to failure mode, selection of the restoration material for endocrown depends on the priority of preserving the tooth or the longevity of the restoration. If the priority is preserving the tooth, resin-modified ceramic and reinforced composite are the choices, while lithium disilicate aims can be selected for the longevity of restoration.

There have been some limitations in this study; differences in pulp chamber morphology could make bias in results. It was an in vitro study, which may differ from a clinical one, where the clinical scanning processing would be less precise because of limitations such as saliva and limited access of the scanner in the oral cavity. Another limitation of this study was the use of a pure tensile test, which doesn’t represent full ranges of forces that occur in clinical situations and likely contribute to endocrown debonding. However, the pure tensile testing was used because it has been adopted in other studies and could allow comparison of these results with previous investigations^25,26,42^. Further studies are needed to investigate the mechanical and adhesive properties of the materials used in and even long-term follow-up sessions in the clinic.

## Conclusions

Based on the findings of this in vitro study, the following conclusions were drawn:

1. Lithium disilicate specimens showed higher tensile force values after thermocycling than other materials used, which may have a promising impact on the survival of endocrown restorations.
2. CAD-CAM reinforced composite showed the lowest tensile force values after thermocycling and presented low mechanical properties in failure mode.

## Data Availability

The datasets generated and/or analysed during the current study are not publicly available due to [the research is not published yet] but are available from the corresponding author on reasonable request.
